# The roles of G protein‐coupled receptor kinase 2 in renal diseases

**DOI:** 10.1111/jcmm.70154

**Published:** 2024-10-22

**Authors:** Jiayin Du, Xiaoyan Wu, Lihua Ni

**Affiliations:** ^1^ Department of Nephrology Zhongnan Hospital of Wuhan University Wuhan Hubei China; ^2^ Department of General Practice Zhongnan Hospital of Wuhan University Wuhan Hubei China

**Keywords:** GPCR, GRK2, GRK2 inhibition, kidney injury, renal diseases

## Abstract

G protein‐coupled receptor (GPCR) kinase 2 (GRK2) is an integrative node in many signalling network cascades. An emerging study indicates that GRK2 can interact with GPCRs and non‐GPCR substrates in both kinase‐dependent and ‐independent modes. Alterations in the functional levels of GRK2 have been found in a variety of renal diseases, such as hypertension‐related kidney injury, sepsis‐associated acute kidney injury (S‐AKI), cardiorenal syndrome (CRS), acute kidney injury (AKI), age‐related kidney injury or hyperglycemia‐related kidney injury. Abnormal GRK2 expression contribute to the development of renal diseases, making them promising molecular targets for treating renal diseases. Blocking the prostaglandin E_2_ (PGE_2_)‐EP1‐Gaq‐Ca^2+^ signal pathway in glomerular mesangial cells (GMCs) by internalizing prostaglandin E_2_ receptor 1 (EP1) with GRK2 may be a potential treatment for diabetic nephropathy (DN). In addition, GRK2 inhibition may have therapeutic effects in a variety of renal diseases, such as SLE‐related kidney injury, DN, age‐related kidney injury, hypertension‐related kidney injury, and CRS. However, there is still a long way to go for the large‐scale application of GRK2 inhibition in the field of renal diseases. In this review, we discuss recent updates in understanding the role of GRK2 in kidney dysfunction. Furthermore, we explore the potential of GRK2 as a possible therapeutic target for renal pathologies. We believe it will shed light on the future development of small‐molecule inhibitors of GRK, as well as the clinical applications in renal diseases.

## 1 INTRODUCTION

Kidney disease is a common non‐communicable disease with a global prevalence that surpasses cardiovascular diseases, cancer, chronic respiratory diseases and diabetes. It affects over 750 million persons worldwide.^1^ Reducing the burden of kidney disease is an important public health concern because of both the number of people affected and the high cost of care. Some of the findings are noteworthy. GRK2, an important subtype of GRKs, is closely associated with impairment in renal dopamine D1 receptor function.^2^ In a mouse model of systemic lupus erythematosus (SLE), both genetic deficiency and pharmacological inhibition of GRK2 can mitigate histopathological alterations in the kidney.^3^ Links between GRK2 and histopathological alterations in the kidney suggest potential therapeutic strategies to prevent kidney disease progression and portray GRK2 as a promising therapeutic target.

The history of GRKs research began with rhodopsin kinase, called GRK1 now and found to specifically phosphorylate light‐activated G‐protein‐coupled receptors rhodopsin. This phosphorylation partially reduced the ability of rhodopsin to interact directly with transducin and significantly increased its ability to interact with the protein arrestin, an inhibitory factor of GPCR signal transduction.^4^ During the development of the study, GRKs constituted a family of seven serine/threonine protein kinases that specifically recognized and phosphorylated agonist‐activated GPCRs, which initiated uncoupling from heterotrimeric G proteins and receptor internalization.^5^

GRK2 was initially recognized as the enzyme that phosphorylated the β‐adrenergic receptor (βAR) in an agonist‐dependent manner, while it had been shown to mediate desensitization of a variety of receptors, such as mu‐opioid receptors,^6^ metabotropic glutamate receptors^7^ and oxytocin receptor.^8^ Besides, emerging evidence indicated that GRK2 could make a difference via a non‐GPCR substrate or non‐phosphorylated pathway.^9^ Hildreth and his colleagues found GRK2 phosphorylated PDGF‐Rβ, a kind of receptor tyrosine kinases, and reduced receptor activity without altering its down‐regulation in 2004.^10^ Naga Prasad proposed GRK2 facilitated PI3K recruitment to the membrane upon agonist stimulation, thus contributing to receptor endocytosis and desensitization in 2002.^11^ In short, GRK2 is a versatile protein that plays a central, integrative role in signal transduction cascades.^12^ As a result, GRK2 participates in the modulation of multiple cellular functions, such as cell proliferation, survival, motility, metabolic homeostasis, inflammation, or angiogenic processes. GRK2 has been demonstrated to finely regulate chemokine‐dependent signalling in lymphocytes and neutrophils during inflammation by the way to trigger desensitization of a variety of chemokine receptors.^13^

GRK2 has also been found to play a crucial role in renal mechanisms of homeostasis. The genetic knockdown of GRK2 using small hairpin interfering RNA (shRNA) caused the mice to present with reduced kidney size, nephrogenesis and glomerular count, impaired glomerular filtration and spontaneously hypertensive.^14^ Mean blood urea was 28.0 in 6‐month‐old mice with GRK2 knockdown using shRNA and 22.7 in controls (*n* = 6). Another research found GRK2 was involved in regulating membrane protrusion and motility in Madin‐Darby canine kidney epithelial cells.^15^ The number of studies on GRK2 in the field of kidney diseases is not as large as that in the field of cardiovascular diseases, but it has been gradually improved in recent years. For instance, researchers have focused on the mechanism of GRK2 in hypertension‐related kidney injury,^16^ S‐AKI^17^ and CRS.^18^ However, a systematic summary and logical sorting of these studies are lacking. Therefore. In order to facilitate the research of GRK2 in the field of kidney diseases and promote its clinical application in the treatment of kidney diseases, this review will systematically summarize the current state of research on the roles of GRK2 in renal diseases.

## 2 ROLES OF GRK2 IN RENAL DISEASES

### 2.1 Hypertension‐related kidney injury

It is acknowledged that GRK2 can exert a powerful inhibitory effect by phosphorylation in vivo environments. It has been shown to inhibit the activity of two substances in renal cells, which can lead to hypertension.

The first, GRK2 can phosphorylate dopamine D1 receptors and thus disrupt the function of sodium transporters leading to hypertension. Dopamine plays an important role in body sodium and BP homeostasis, especially during states of increased sodium intake. Dopamine can lead to natriuresis and diuresis by activating D1 receptors in renal proximal tubules (PT) and subsequently inhibiting the activity of sodium transporters such as sodium hydrogen exchangers and Na, K‐ATPases. About 60% of sodium excretion during a moderate salt load is mediated by the activation of renal D1 receptors.^19^ Moreover, among the D1‐like receptors expressed in PT of human, only D1 (but not D5) receptors contribute to inhibition of sodium and water reabsorption by dopamine.^20^ Like most GPCRs, GRK‐mediated phosphorylation reaction can lead to their desensitization. But the more important question is what triggers this phosphorylation. Many studies have found that the answer is oxidative stress and had elucidated the signal transduction. It was confirmed that prenatal Lipopolysaccharide exposure, via an increase in oxidative stress, impaired renal D1R function and led to hypertension in the offspring.^21^ Another study indicated that maternal diabetes mellitus‐programed hypertension in the offspring was caused by impaired renal D1 receptor function because of oxidative stress that was mediated by increased PKC‐GRK2 activity.^22^ Oxidative stress increases protein kinase C (PKC) activity,^2^ which is associated with redox‐sensitive nuclear transcription factors, NF‐κB. NF‐κB is activated in conditions associated with oxidative stress. In a study, the treatment of rats with BSO, an oxidant, increased nuclear translocation of NF‐κB, which was accompanied by increased PKC expression. Moreover, tempol, which could decreased oxidative stress, while abolishing NF‐κB translocation normalized PKC expression and activity.^16^ Therefore, oxidative stress activates NF‐κB, which causes increased PKC expression, thereby increasing its activity. After this, GRK‐2 can be phosphorylated by PKC, which leads to its membrane translocation in PT and increased activity. In the next moment, GRK2 leads to renal D1 receptor hyperphosphorylation, which impairs the coupling of the D1 receptors and G proteins, leading to impaired signal transduction and loss of inhibition of sodium transport.^23^ The inability of renal D1 receptors to inhibit the activity of sodium hydrogen exchangers and Na, K‐ATPases and promote sodium excretion, which triggers by oxidative stress, may contribute to an increase in blood pressure.

The second, GRK2 can phosphorylate ubiquitin protein ligases and thus disrupt the function of sodium transporters leading to hypertension. This time, epithelial Na^+^ channels (ENaCs) are the sodium transporters involved. ENaCs mediate the transport of sodium across epithelia in the kidney and are required for blood pressure regulation. They are inhibited by ubiquitin protein ligases, such as Nedd4 and Nedd4‐2. Loss of this inhibition leads to hypertension.^24^ A study reported that GRK2 interacted with both Nedd4 and Nedd4‐2 and it was capable of phosphorylating both Nedd4 and Nedd4‐2 at multiple sites.^25^ This result supported and extended the role of GRK2 in sodium transport regulation. It is also stated that GRK2 has a good chance of inactivating Nedd4 and Nedd4‐2 by phosphorylation, which in turn leads to the overactivation of ENaCs and hypertension.

In addition, GRK2 has also been found to play a role in renal vascular reactivity impairment caused by hypertension. Peroxisome proliferator‐activated receptors (PPARs) are ligand‐activated transcription factors that belong to the nuclear receptor superfamily. PPARγ, one subtype of PPARs, is expressed in vascular smooth muscle cells.^26^ PPARγ is reported to have the function of renal vascular reactivity improvement, and its effect in the kidney extends beyond glycemic and lipidemic control.^27^ At the same time, numerous studies demonstrated the possibility that PPARγ transcriptionally regulated the regulators of G protein signalling (RGS) molecules.^28^ GRK2 is also an RGS family of protein. Importantly, the research showed that PPARγ could activate Raf/MEK/ERK1/2 signalling pathways where activated ERK1/2 reduced GRK2 activity^29^ (Abbreviations: Raf, mitogen activated protein kinase kinase kinase; MEK, mitogen activated protein/ERK kinase; ERK, extracellular signal‐regulated protein kinase). A study based on spontaneously hypertensive rats revealed a possible mechanism of hypertension‐related kidney injury: hypertension resulted in the downregulation of PPARγ and subsequent upregulation of GRK2, which impaired renal vascular reactivity.^30^

**FIGURE 1:**
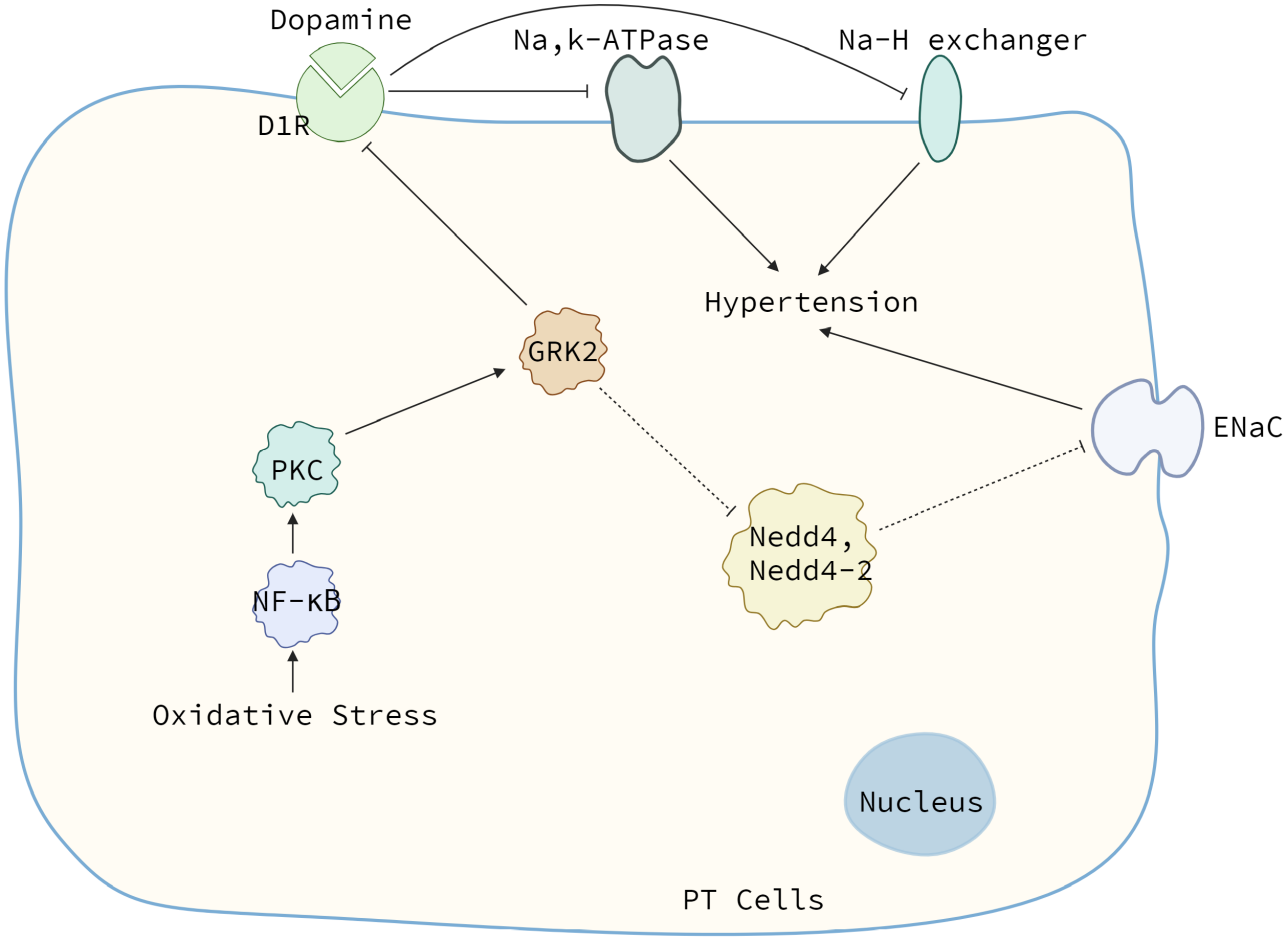
GRK2 and hypertension‐related kidney injury. Oxidative stress generates the elevation of NF‐κB‐PKC‐GRK2 signalling, which impairs the function of D1R in PT cells. The inability of D1R to inhibit Na, K‐ATPase or Na‐H exchanger activity and promote sodium excretion contributes to an increase in blood pressure. In addition, ENaC in PT cells is inhibited by ubiquitin protein ligases, such as Nedd4 and Nedd4‐2. Loss of this inhibition due to inactivation of Nedd4 and Nedd4‐2 by GRK2 leads to hypertension. D1R, dopamine D1 receptor; ENaC, epithelial Na^+^ channel; PKC, protein kinase C; PT, proximal tubule.

In general, GRK2 can break the balance of sodium transport by interfering the function of sodium hydrogen exchangers, Na, K‐ATPases or ENaCs and subsequently causes renal damage leading to hypertension (Figure 1). And the upregulation of GRK2 related to hypertension can impair renal vascular reactivity.

### 2.2 Sepsis‐associated acute kidney injury

Sepsis is defined as a life‐threatening organ dysfunction caused by an exaggerated immune response of the host to infection. One of the organs most affected by sepsis is the kidney, which may result in S‐AKI.^31^ Then a mechanism relative to GRK2 will be introduced, which may help to explain S‐AKI. Septic shock is characterized by hypotension, decreased systemic vascular resistance and impaired vascular reactivity. But the kidney presents a paradoxical vasoconstriction during sepsis. It keeps its ability to respond to the endogenous vasoconstrictors released due to sepsis‐induced hypotension. Thus, the vascular tonus in the kidney is maintained or even increased during sepsis,^32^ which contributes to acute renal failure.^33^ What causes this peculiarity of the kidney?

NO production is increased by the expression of the enzyme nitric oxide synthase isoform 2 (NOS‐2) in the kidney during sepsis in response to inflammatory cytokines.^34^ Based on data from a study, both GRK2 mRNA and protein levels were reduced in the kidney of septic mice. In addition, NOS‐2 selective inhibitor increased GRK2 levels in the septic kidney, and GRK2 levels of NOS‐2‐KO mice were not reduced during sepsis, thus confirming that NO was indeed involved in the kidney GRK2 disappearance. And then the study also found that sepsis increased α1 adrenergic receptor density in the kidney and sepsis‐induced reduction in renal blood flow was blocked by prazosin treatment. Therefore, the NO‐dependent reduction in GRK2 level in the kidney, which may lead to a reduced phosphorylation and internalization of the α1 adrenergic receptors, leads to the maintenance of a normal α1 adrenergic receptor density.^17^ α1 adrenergic receptors are abundant in kidney tubules, and the effects due to their activation interfere with renal vascular tone. The preservation of the density and/or functionality of the receptor in the kidney may lead to a vasoconstriction. In the meantime, the concentration of vasoconstrictor mediators increases in the kidney of Sepsis.^35^ The increased concentration of vasoconstrictor mediators together with the preservation (and even increase) of the response to them may help to explain S‐AKI.

**FIGURE 2:**
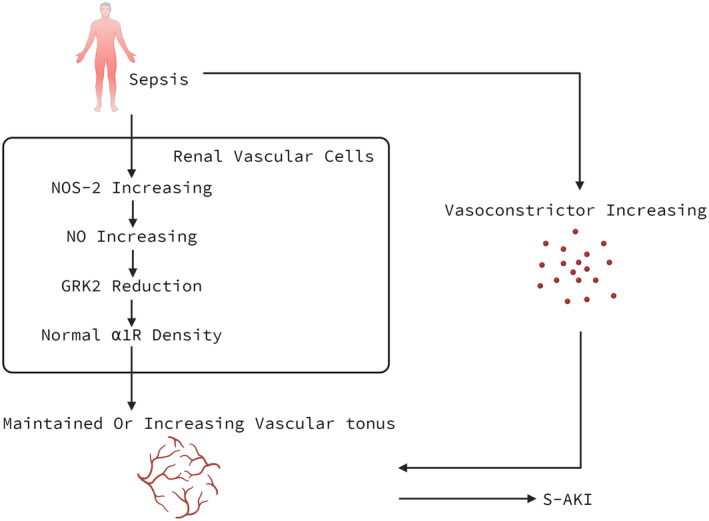
GRK2 and S‐AKI. A NO‐dependent reduction in GRK2 level during sepsis leads to the maintenance of a normal α1 adrenergic receptor density in renal vascular cells. In the meantime, the concentration of vasoconstrictor mediators, such as norepinephrine and renin, increases in the kidney of Sepsis. Under the influence of these two aspects, the vascular tonus in the kidney is maintained or even increased during sepsis, which contributes to S‐AKI. NOS‐2, the enzyme nitric oxide synthase isoform 2; S‐AKI, sepsis‐associated acute kidney injury.

In conclusion, a NO‐dependent reduction in GRK2 level in the kidney of Sepsis plays a role in S‐AKI by interacting with α1 adrenergic receptors (Figure 2).

### 2.3 Cardiorenal syndrome and acute kidney injury

GPCR signalling is a crucial regulator of cardiovascular and renal function. Ligand binding to an extracellular active site of the receptor induces a conformational change in the GPCR, which allows for coupling with heterotrimeric guanine‐nucleotide regulatory proteins (G‐proteins).^36^ G‐proteins are heterotrimers of α, β and γ subunits known as Gα, Gβ and Gγ, respectively. Receptor activation facilitates the exchange of GDP for GTP on the Gα subunit that results in dissociation of the Gα from the Gβγ. Dissociated Gα subunit signal via activation of an effector molecule to produce second messengers, which modulate a variety of downstream processes.^37^ Under basal conditions, GRK2 is distributed primarily in the cytoplasm. Upon GPCR activation, GRK2 is translocated to the plasma membrane via binding with the activated Gβγ subunits. GRK2‐mediated phosphorylation of the GPCR causes β‐arrestin recruitment to the receptor and consequent inhibition of dissociated G‐protein from coupling to the receptor/β‐arrestin complex and further attenuation of downstream signalling.^38^ Further, the potential of GPCR‐Gβγ‐GRK2 signalling as a possible mechanism of CRS will be discussed below.

**FIGURE 3:**
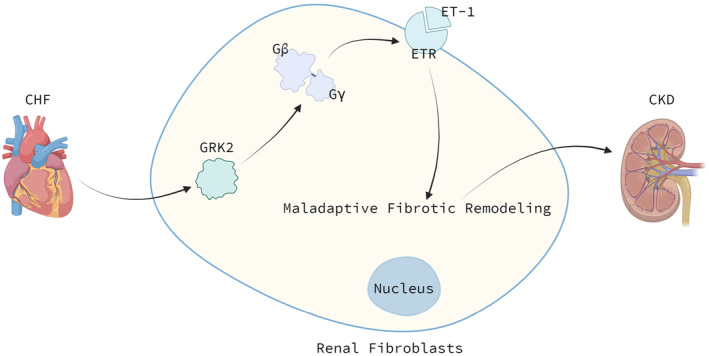
GRK2 and CRS. CHF generates the elevation of Gβγ‐GRK2 signalling and the following overactivation of ETR function in renal fibroblasts. This leads to maladaptive fibrotic remodelling in cells, which facilitates the formation of CKD. CHF, chronic heart failure; CKD, chronic kidney injury; CRS, cardiorenal syndrome; ET‐1, endothelin‐1; ETR, endothelin receptor.

CRS describes a specific acute and chronic clinical picture in which the heart or the kidney is primarily dysfunctional and secondarily affect each other. CRS type 2 (CRS2) is characterized as chronic heart failure (CHF) accompanied by the development of chronic kidney injury (CKD).^39^ To recapitulate the clinical features of CRS2 progression, a study used a non‐ischemic transverse aortic constriction (TAC) mouse model of pressure‐overload to induced HF. By 12 weeks after TAC, mice had developed CKD secondary to CHF, reflected by elevated serum creatinine levels, emerged morphological and molecular signs of tubular damage and increased focal tubulo‐interstitial and perivascular fibrosis in the kidneys.^40^ Furthermore, authors detected the elevation of Gβγ‐GRK2 signalling in kidneys at 12 weeks post‐TAC, which resulted in the elevated level of endothelin (ET)‐1 along with increased protein expression and membrane localization of ET receptors (ETA and ETB). Upregulation of both ET and Gβγ‐GRK2 signalling was attenuated by small‐molecule Gβγ inhibitor gallein treatment. ET‐1 receptors, a kind of GPCRs, are widely expressed in the kidney.^41^ ET‐1 is a potent vasoconstrictor peptide with pro‐inflammatory, mitogenic, and pro‐fibrotic properties that is closely involved in both normal renal physiology and pathology. Increasing evidence indicates that the ET system is involved in an array of renal disorders.^42^ Maladaptive fibrotic remodelling led by the elevated level of ET‐1 is a key component of CKD. In summary, CHF generates the elevation of Gβγ‐GRK2 signalling, the following ET system activation in renal fibroblasts and maladaptive kidney remodelling, which facilitates the formation of CKD (Figure 3).

Likewise, the elevation of Gβγ‐GRK2 signalling and the following ET system activation also play a role in AKI. To determine the role of Gβγ‐GRK2 signalling in kidney dysfunction besides the crosstalk with the heart, a direct bilateral ischemia reperfusion (I/R) AKI model was also implemented. Importantly, Gβγ‐GRK2, ET‐1 and ETA gene expression was elevated in the kidneys of AKI I/R mice.^43^ And pretreatment with a physiologic dose of gallein, a Gβγ‐GRK2 interaction inhibitor, was renoprotective.^18^ In this model, gallein attenuated renal dysfunction, tissue damage, fibrosis, inflammation and ET system activation. Overall, these data support a direct role for GPCR‐Gβγ‐GRK2 in both CRS and AKI.

### 2.4 Age‐related kidney injury

Numerous studies have shown that the aging process has caused many structural and functional changes in the kidney.^44^ Dopamine fails to inhibit proximal tubular Na, K‐ATPase activity and to promote sodium excretion in old rats. This is due to reduced D1 receptor expression and binding sites and defective D1 receptor‐G protein coupling resulting from hyperphosphorylation of D1 receptors in the renal proximal tubules of these animals.^45^ In addition, an increase in oxidative stress is associated with aging, which leads to morbidity and mortality.^46^ The above account suggests that oxidative stress and defective D1 receptor function coexist within the aging kidney. A study further verified the correlation: oxidative stress caused the age‐related decline in dopamine D1 receptor function.^47^ Oxidative stress associated with aging generates elevated NF‐κB‐PKC‐GRK2 signalling, which in turn leads to an age‐related decline in dopamine D1 receptor function in PT cells.^2^

Then GRK2 may be involved in the formation of age‐related kidney injury in males by another mechanism. A decline in baroreflex buffering of blood pressure occurs as human males age and is due, at least in part, to a reduction in alpha‐1 adrenergic receptor function.^48^ A study found that the reduction of renal interlobar arterial constriction had an early onset in male rats compared to female rats, which coincided with the gender‐specific downregulation of renal vasoconstrictor ability and renal function in humans. And the declines in alpha‐1 adrenergic receptor binding and G alpha q expression and also the increases in GRK2 and tyrosine phosphatases expression likely related to the age‐related decline of vasoconstriction in the kidney of male rats. But similar results were not found in female mice. The information that the expression of alpha‐1 adrenergic receptors is greater in female rats than males and GRK2 expression does not increase during aging could relate to the gender differences in vasoconstrictor function with aging. So, with aging in males, the increase in GRK2 expression may result in the decline of vasoconstriction in the kidney by inhibiting alpha‐1 adrenergic receptor function.^49^

### 2.5 Hyperglycemia‐related kidney injury

Hyperglycemia associated with diabetes plays an important role in the generation of reactive oxygen species (ROS), leading to increased oxidative stress. Furthermore, in diabetes, sodium retention coexists with increased oxidative stress.^50^ A study tested the relationship between the two and concluded that oxidative stress associated with hyperglycemia caused an increase in activity and expression of PKC, which increased GRK2 translocation to the proximal tubular membrane, subsequent phosphorylation of the D1‐like receptors and their uncoupling from G proteins and loss of responsiveness to agonist stimulation.^51^ Besides, insulin can play a similar role to the oxidative stress described above. Insulin therapy is the most important treatment aspect of diabetes mellitus. However, hyperinsulinemic animals and renal cell cultures treated with insulin showed reduced D1R number and defective D1Rs coupling to G proteins.^52^ A study investigated insulin‐mediated D1R desensitization and underlying molecular mechanisms in opossum kidney (OK) cells. Insulin activated the PI3K‐PKC‐GRK2 cascade, causing D1R serine phosphorylation, which led to D1R downregulation and uncoupling from G protein and resulted in the failure of D1R agonist to stimulate G protein and inhibited Na‐K‐ATPase activity.^53^ In brief, upregulation of GRK2 is involved in D1R desensitization induced by oxidative stress or insulin treatment in patients with hyperglycemia.

In addition, GRK2 may be an important contributor to renal vascular endothelial dysfunction in diabetes. Human umbilical venous endothelial cells (HUVECs) were exposed to high glucose and high insulin (HG/HI) to mimic insulin‐resistant diabetic conditions.^54^ GRK2 expression and membrane translocation were upregulated under HG/HI conditions. GRK2, which was upregulated by HG/HI, led to a tonic inhibition of the insulin Akt/eNOS pathway in endothelial cells (Akt and endothelial NO synthase (eNOS) were insulin downstream effectors). These findings suggested that GRK2 was an important negative regulator of insulin signalling in human vascular endothelial cells and might be key to the vicious cycle of insulin resistance disease. Moreover, insulin resistance is associated with endothelial dysfunction. It has been known that dysfunction of vascular endothelial cells in insulin‐resistant states could be explained by alterations in insulin intracellular signalling that affect the production of NO.^55^ A study found that the negative regulation by vascular GRK2 of Akt and eNOS led to the impairment of aortic endothelium‐dependent relaxations in the ob/ob mouse.^56^ These findings also provided new insights into the pathogenesis of diabetes‐associated vascular endothelial dysfunction. Elevated GRK2 contributes to the development of insulin resistance and associated vascular endothelial dysfunction in diabetic patients. GRK2 could be a potential target in the prevention and treatment of vascular disorders in insulin‐resistant individuals.

## 3 THERAPEUTIC POTENTIALS OF GRK2 IN RENAL DISEASES

The up or downregulation of GRK2 is closely related to plentiful pathological disorders. GRK2 upregulation can exacerbate cardiac ischemia; moreover, elevated kinase levels occur in the early stages of heart failure (HF) and in hypertensive individuals. Lymphocyte GRK2 protein level can even independently predict prognosis in patients with HF.^57^ GRK2 upregulation can cause alterations in the insulin signalling cascade, which can result in insulin resistance. Increased GRK2 levels are also associated with the degree of cognitive impairment that is typically observed in Alzheimer's disease. So GRK2 is currently a well‐established therapeutic target for the treatment of cardiac ischemia, HF, hypertension, insulin resistance and Alzheimer's disease.^58^ As GRK2 is ubiquitously distributed in the human kidney, its therapeutic potential in a variety of renal diseases cannot be ignored either.

The therapeutic potential of GRK2 in DN had attracted much attention among the renal diseases. DN is a major microvascular complication of diabetes mellitus, which is characterized by complete or partial microalbuminuria, GMC proliferation with deposition of extracellular matrix (ECM) at the glomerular level and global glomerular sclerosis and atrophy. It is the most frequent cause of end‐stage renal diseases with no definitive therapy available so far.^59^ There is increasing awareness and consensus that the abnormal growth and activity of GMCs play an important role in the pathophysiology of the early stages of DN. PGE_2_‐EP1‐Gaq‐Ca^2+^ signal pathway exists in GMCs, and their overactivation is related to the formation of DN. Excessive PGE_2_ has been shown to be associated with an increased glomerular filtration rate in the early stage of DN and with subsequent glomerular hypertrophy, proteinuria and renal injury.^60^ This is because many different functions of GMCs are controlled by intracellular Ca^2+^ concentration. Under the overactivation of PGE_2_‐EP1‐Gaq‐Ca^2+^ signal pathway, GMC changes their phenotype and becomes proliferative and matrix expanding subsequently, which promotes ECM accumulation, mesangial hypertrophy and even glomerulosclerosis and ultimately accelerates the progression of DN. A study found Berberine (BBR) decreased the abnormal concentration of Ca^2+^, the increased levels of PGE_2_, the high expression of EP1 and Gaq and suppressed the proliferation of GMC in DN. Importantly, increased expression levels of GRK2 and β‐arrestin2 coincided with the decreased fluorescence intensity of EP1 on the GMC membrane. It proved that the internalization of EP1 was modulated by GRK2 and β‐arrestin2.^61^ Therefore, internalizing EP1 with GRK2 to block PGE_2_‐EP1‐Gaq‐Ca^2+^ signal pathway and subsequently suppress the proliferation of GMC may be a potential treatment for DN. And BBR could be a promising anti‐DN medicine in the future because of its renoprotective effects via regulating the PGE_2_‐EP1‐Gaq‐Ca^2+^ signal pathway.

Selective GRK2 inhibition might be an innovative therapeutic strategy to treat renal diseases in which GRK2 overexpression leads to dysregulated signalling pathways.^62^ On one hand, GRK2 inhibitors (paroxetine (PAR) and CP‐25) attenuate histopathological alterations in SLE kidneys by suppressing plasma cell differentiation. SLE is a chronic autoimmune disease affecting multiple organs, characterized by dysregulated B cells and autoantibody overproduction. Overactivated B cells, recognized as the primary source of autoantibodies, are central to the pathogenesis of SLE. B lymphocyte‐induced maturation protein 1 (Blimp1) and interferon regulatory factor 4 (IRF4) are pivotal transcription factors that control the differentiation of plasma cells from autoreactive B cells. The increased size and swollen state of glomeruli, inflammatory cells infiltration, glomerular cell proliferation, immune complex deposition, thickened basement membrane, and hyaline thrombi in glomeruli were found in SLE mice, which could be significantly ameliorated by GRK2 inhibitors (PAR and CP‐25) administration. Inhibition of GRK2 suppressed plasma cells differentiation and alleviated pristane‐induced SLE by reducing Blimp1 and IRF4 expression.^3^ On the other hand, paroxetine‐mediated GRK2 downregulation may be a potential therapeutic strategy for DN. After analysing the DN single‐nucleus transcriptome sequencing and Connectivity Map (CMap) database, cell‐based experiments, animal experiments and histopathological assessment, PAR was found to has excellent podocyte protection effects, reduce levels of proteinuria and ameliorate the tissue damage in DN. Mechanistically, PAR may reduce inflammation and oxidative stress in kidney tissue through the inhibition of GRK2, which in turn inhibits the key inflammatory regulators p‐p65 and NLRP3 and restores the endogenous NRF2 antioxidant pathway^63^ (Table 1).

**TABLE 1:** GRK2 inhibitors in the field of renal disease treatment.

GRK2 inhibitors	Model or method	Mechanism	Renal disease
CP‐25 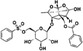	Pristane‐induced SLE mice model	Blimp1 and IRF4 expression↓; plasma cells differentiation↓	Kidney damage in SLE^3^
PAR 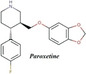			
PAR 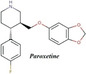	Method: single‐cell transcriptome sequencing and CMap database; Cell: Immortalized human podocyte; DKD model: intraperitoneal injection of STZ to C57BL/6J mice	p‐p65 and NLRP3↓; endogenous NRF2 antioxidant pathway↑	DN^63^

Abbreviations: Blimp1: B lymphocyte‐induced maturation protein 1; CMap: Connectivity Map; DN: diabetic nephropathy; IRF4: interferon regulatory factor 4; PAR: paroxetine; SLE: systemic lupus erythematosus.

Above all, there are great potentials for researchers to develop selective GRK2 inhibitors to treat renal diseases. As for age‐related kidney injury, the upregulation of GRK2 contributes to the age‐related decline in dopamine D1 receptor function and the reduction in alpha‐1 adrenergic receptor function with aging in male. A study found that suppression of GRK2 expression by inhibition of early‐growth response 1 (EGR‐1) binding to GRK2 might be a promising approach to mitigate adrenergic desensitization.^64^ Consequently, GRK2 inhibition could possibly ameliorate the age‐related decline in renal function and reduce the need for renal dialysis. As for hypertension‐related kidney injury, an increase in GRK2 protein expression in lymphocytes from hypertensive patients, presumably due to the desensitization of β adrenergic receptors that mediated vasodilatation, had been found.^65^ This finding suggested that inhibition of GRK2 could represent a viable therapeutic strategy for the treatment of hypertension‐related kidney injury.^66^ As for CRS, the elevation of Gβγ‐GRK2 signalling in kidneys led by CHF is a key component of CKD, as mentioned above. Taking the bidirectional nature of the crosstalk between heart and kidney into consideration, Polhemus and colleagues found radiofrequency renal denervation (RF‐RDN) protected the ischemic heart via inhibiting GRK2 and increasing NO signalling.^67^ RF‐RDN, which inhibited activity of renal sympathetic efferent and afferent nerves that lay within and immediately adjacent to the wall of the renal artery, served to reducing the interference of the kidney on the heart in the study. So, both the heart and kidney cause damage to each other by increasing GRK2. Further exploration of inhibiting the GPCR‐Gβγ‐GRK2 signalling pathway might lead to the development of novel approaches for CRS treatment.

Drug selectivity is an important and challenging difficulty in renal disease treatment with GRK2 inhibition. If the selectivity of candidate drugs is not paid enough attention, it may lead to strong side effects and even the toxic effects of drugs on the body are far greater than their therapeutic effects.^68^ The GRK family, the largest family of signal‐transducing proteins already known, is mostly expressed in all tissues throughout the body. And members of this family have a wide range of substrates of action and repeat substrate specificity.^69^ GRK2 and GRK5 have common functions implicated in the regulation of heart failure, though GRK5 has also been involved in diseases like hypertension, cancer, diabetes and Alzheimer's disease. Low selectivity poses a significant safety risk, which makes it necessary to develop low‐toxicity and highly selective inhibitors for GRK2. Among all of the described GRK2 inhibitors, the most active ones are the Takeda compounds 2a and 2b, which also have a good target selectivity, as they are inactive toward other GRK isoforms at concentrations up to 125 μM. Promising results are also obtained with the RNA aptamer 3a, the indazole derivative 5a and small peptide inhibitors. Although advances have been made toward developing inhibitors that are selective for GRK2, safe and effective GRK2 inhibitors for kidney disease still need to be studied by silico investigation and chemical and biological experiments.^70^

## 4 SUMMARY AND PROSPECT

GRK2, a versatile protein, has been shown to mediate desensitization of a variety of receptors by phosphorylation in an agonist‐dependent manner. GRK2 signalling in the human body is an extremely complex network. GRK2 regulates the activity of various receptors expressed on renal cells, which may give rise to hypertension‐related kidney injury, S‐AKI, CRS, AKI, age‐related kidney injury, and hyperglycemia‐related kidney injury. In addition, the potential of GRK2 in the treatment of kidney disease is also beginning to show. Internalizing EP1 with GRK2 to block PGE2‐EP1‐Gaq‐Ca^2+^ signal pathway in GMCs may be a potential treatment for DN. As we know GRK2 is upregulated in various kidney diseases, GRK2 inhibition might be an innovative therapeutic strategy to treat renal diseases, such as SLE‐related kidney injury, DN, age‐related kidney injury, hypertension‐related kidney injury, and CRS.

After sorting out the literature, it is found that the inhibition of D1 receptor activity in PT by GRK2 is a focus of research, which is triggered by oxidative stress induced by various pathological conditions. There exists a defect in renal D1 receptor function in hypertension, diabetes, and aging, conditions that are associated with oxidative stress.^16^ This provides insight into the mechanisms that cause other types of kidney damage. For instance, inflammation and oxidative stress are closely related processes. Chronic inflammation leads to an imbalance between antioxidants and molecules with antioxidant properties.^71^ And inflammation is also a common cause of kidney damage.^72^ Hyperuricemia‐induced autophagy and NLRP3‐dependent inflammation are critically involved in the development of renal damage.^73^ SGLT2 inhibitors reduce inflammation and increase autophagy to alleviate podocyte damage in lupus nephritis.^74^ So, future studies can be designed to test the hypothesis that oxidative stress associated with inflammation activates the PKC‐GRK2 pathway, resulting in loss of D1 receptor function in PT and subsequent inflammation‐related kidney damage.

Many kinds of potent and selective GRK2 inhibitors have been developed, such as the Takeda compounds 2a and 2b and the indazole derivative 5a^62^. Gallein inhibits GRK2 by disrupting Gβγ‐GRK2 binding and can directly alleviate myocardial and renal dysfunction and fibrosis.^75^ In the long run, there appears to be promising research potential regarding the therapeutic impact of these inhibitors on nephropathy caused by elevated GRK2. It is expected to provide new drugs for the treatment of clinical renal diseases. Though partial GRK2 inhibition plays a positive role in the therapy of many renal diseases, total inhibition of GRK2 can have deleterious outcomes on kidney function and development. Therefore, the detrimental outcomes of GRK2 inhibitors on the kidney should be carefully evaluated when they are employed in the therapeutic therapy of renal illnesses.^14^

For the first time, our review summarized the role of GRK2 in renal diseases and provides many feasible entry points for GRK2 research in the field of kidney disease in the future. Limited by the number of studies, much of signalling pathways in this review are based on a single research conclusion or speculation. Thus, a great deal of researches might be planned to give them more direct proof. Further studies are encouraged to bridge the gaps in our understandings the roles of GRK2 in renal disorders.

## AUTHOR CONTRIBUTIONS

**Jiayin Du:** Methodology (equal); project administration (equal); resources (equal); writing – original draft (equal); writing – review and editing (equal). **Xiaoyan Wu:** Funding acquisition (equal); investigation (equal); supervision (equal); writing – original draft (equal). **Lihua Ni:** Conceptualization (lead); resources (equal); software (equal); supervision (equal); writing – original draft (equal); writing – review and editing (equal).

## FUNDING INFORMATION

This work was supported by grants from the National Natural Science Foundation of China (82200807 and 82370696). Outstanding Young and Middle‐aged Talents Training Program of Zhongnan Hospital of Wuhan University(ZNYQ2022007). The Research Fund from Medical Sci‐Tech Innovation Platform of Zhongnan Hospital, Wuhan University (ZNXKPY2022046 and ZNXKPY2022048). The Translational Medicine and Interdisciplinary Research Joint Fund Project (XKJC202313).

## CONFLICT OF INTEREST STATEMENT

The authors declare that they have no competing interests.

## CONSENT FOR PUBLICATION

We confirm that the manuscript has been read and approved by all named authors and that there are no other persons who satisfied the criteria for authorship but are not listed. We further confirm that the order of authors listed in the manuscript has been approved by all of us.

## Data Availability

Not applicable.
